# Comparative efficacy and safety of wenxin granule combined with antiarrhythmic drugs for atrial fibrillation: A protocol for a systematic review and network meta-analysis

**DOI:** 10.1097/MD.0000000000024434

**Published:** 2021-01-22

**Authors:** Shuqing Shi, Yuguang Chu, Qiulei Jia, Yuanhui Hu

**Affiliations:** a Department of Cardiovascular, Guang’anmen Hospital, China Academy of Chinese Medical Sciences; b Graduate School, Beijing University of Chinese Medicine, Beijing, China.

**Keywords:** antiarrhythmic drugs, atrial fibrillation, network meta-analysis, protocol, randomized controlled trials, wenxin granule

## Abstract

**Background:**

The combination of Chinese patent medicine Wenxin Granules (WXG) and antiarrhythmic drugs has been widely used in the treatment of atrial fibrillation (AF), but the results are controversial. This study will conduct a network meta-analysis (NMA) based on data from randomized controlled trials to evaluate the efficacy and safety of WXG combined with ADDs (amiodarone, metoprolol, propafenone, bisoprolol, or other antiarrhythmic drugs) in the treatment of AF, which will perform comparisons or rankings of efficacy among the currently available therapeutic schemes in order to provide evidence to determine the optimal threshold and treatment regimen to AF patients.

**Methods and analysis:**

A comprehensive systematic literature search will be conducted in Cochrane Library, PubMed, Web of Science, EMBASE, Chinese Biomedical Literature Database (SinoMed), Chinese National Knowledge Infrastructure (CNKI), and WanFang database for randomized controlled trials about the WXG with ADDs. The NMA will be conducted following the PRISMA-NMA guidelines. Statistical analyses will be conducted by using Stata software (version 14.0) and RevMan software (version 5.3).

**Results:**

The results of this NMA will provide a high-quality evidence for the efficacy of WXG combined with ADDs in the treatment of AF, and a ranking of the therapeutic classes will also be presented.

**Conclusion:**

The protocol will provide updated evidence for the application of WXG for AF.

## 1 Introduction

Atrial fibrillation (AF) is the most common arrhythmia in clinical practice.^[1–2]^ The main clinical features of AF are the high-frequency electrical activity of the atria, asynchronous atrial contractions and irregular ventricular excitement.^[2]^ In 2010, the number of men and women suffering from AF worldwide was as high as 20.9 million and 12.6 million, respectively.^[3–4]^ In Europe and the United States, the proportion of adults over 40 years of age who develop AF can reach a quarter.^[5–7]^ It is estimated that by 2030, the number of patients with AF in Europe will be approximately 17 million, and there will be 1.2 to 2.1 million newly diagnosed AF patients each year.^[8]^ AF has become a major public health problem that affects the health of national residents, which hinders social and economic development. The prevention and treatment of AF has become one of the main battlefields in the field of cardiovascular disease.

Current treatments for AF include radiofrequency ablation, conventional surgery, and drug therapy. In 2014, the guidelines issued by the American Heart Association/American College of Cardiology/American Heart Rate Association (AHA/ACC/HRS) recommended that patients with symptomatic AF use at least 1 class I or class III antiarrhythmic drugs after they are ineffective and recommend catheterization Radiofrequency ablation (Class I recommendation).^[9]^ The results of many clinical randomized controlled trials show that supplementary and alternative drugs have similar antiarrhythmic effects with AAD, and the incidence of adverse events is low.^[10–11]^ Among them, Wenxin granule (WXG), which is composed of 5 kinds of Chinese herbal medicines, including *Ginseng* (ren shen), *Polygonatum* (huang jing), *pseudo-ginseng* (san qi), *lamber* (hu po), and *Nardostachys Chinensis* (gan song), is commonly used in AF treatment practice. The formula of WXG is based on the traditional Chinese medicine theories of tonifying Qi, nourishing Yin, promoting blood circulation to remove the obstructions to calm the mind.

There are many clinical studies on the treatment of AF with WXG, but there is still a lack of comprehensive collation and systematic evaluation of the efficacy and safety of WXG intervention in AF. Therefore, the purpose of this study is to conduct a comprehensive and systematic evaluation of the effectiveness and safety of WXG combined with AADs in the treatment of AF.

## 2 Material and methods

### 2.1 Study registration

We will conduct this network meta-analysis of clinical trials in conformity with the guidelines put forth by the Preferred Reporting Items for Network Meta- (PRISMA-NMA) statement,^[12]^ and we had registered the protocol on the PROSPERO International (No. CRD42020163601).

### 2.2 Data sources and search strategy

In this study, we will search 7 commonly used databases: Cochrane Library, PubMed, Web of Science, EMBASE, Chinese Biomedical Literature Database (SinoMed), Chinese National Knowledge Infrastructure, and WanFang database. The retrieval time begins on the database's build date and ends in November 2020. In addition, the reference documents of the included studies will be traced back to supplement the acquisition of relevant documents. The search strategy will be adjusted according to the characteristics of different databases. Table 1 shows the details of the search strategy in PubMed.

**Table 1:** Pubmed search strategy.

No.	Search items
#1	(((((((((Atrial Fibrillations [Title/Abstract]) OR Fibrillation, Atrial [Title/Abstract]) OR Auricular Fibrillation [Title/Abstract]) OR Fibrillation, Auricular [Title/Abstract]) OR Persistent Atrial Fibrillation [Title/Abstract]) OR Familial Atrial Fibrillationl [Title/Abstract]) OR Paroxysmal Atrial Fibrillation [Title/Abstract]) OR Atrial Fibrillation, Paroxysmal [Title/Abstract])) OR “Atrial Fibrillation”[Mesh]
#2	(((((“wenxin granule”[Title/Abstract]) OR (“wenxin keli”[Title/Abstract])) OR (“wenxin granules”[Title/Abstract])) OR (“wenxin formula”[Title/Abstract])) OR (WXG[Title/Abstract])) OR (“wenxin”[Title/Abstract])
#3	(((“amiodarone”[Title/Abstract]) OR (“metoprolol”[Title/Abstract])) OR (“propafenone”[Title/Abstract])) OR (bisoprolol [Title/Abstract])
#4	(Randomized Controlled Trial [Publication Type]) OR randomized [Title/Abstract]
#5	#1 AND #2 AND #3 AND #4

### 2.3 Inclusion and exclusion criteria

#### 2.3.1 Types of studies

Randomized controlled trials regarding WXG combined with AADs (amiodarone, metoprolol, propafenone, bisoprolol, or other AADs) for AF will be included. There are no restrictions on the patient's previous disease, course of disease, age, gender, and race.

#### 2.3.2 Types of participants

Patients who have undergone an ECG or 24-hour Holter examination and meet the AF diagnostic criteria (aged 18 and over, with no upper age limit) of AHA/ACC/HRS 2019 Atrial Fibrillation Treatment Guidelines^[13]^ will be included.

#### 2.3.3 Types of interventions

On the basis of conventional treatment, the experimental group will be treated with WXG combined with AADs (amiodarone, metoprolol, propafenone, bisoprolol, or other AADs). Conventional treatments include antiplatelet agents, β-receptor blockers, renin-angiotensin aldosterone inhibitors, blood lipids and blood pressure regulating drugs, and symptomatic treatment drugs for patients with other diseases.

#### 2.3.4 Types of comparison

Patients in the control group are those who only have received AADs treatment without WXG.

#### 2.3.5 Types of outcomes

The primary outcomes will be the clinical efficacy, clinical symptom efficacy, conversion success rate, sinus rhythm maintenance rate, number of AF episodes, P wave dispersion, and P wave maximum time.

The secondary outcomes will be the indicators of cardiac function, such as left ventricular ejection fraction (LVEF), left ventricular end diastolic diameter (LVEDD), left atrial diameter (LAd), ventricular rate, and the occurrence of adverse reactions.

### 2.4 Studies selection and data extraction

**Figure 1:**
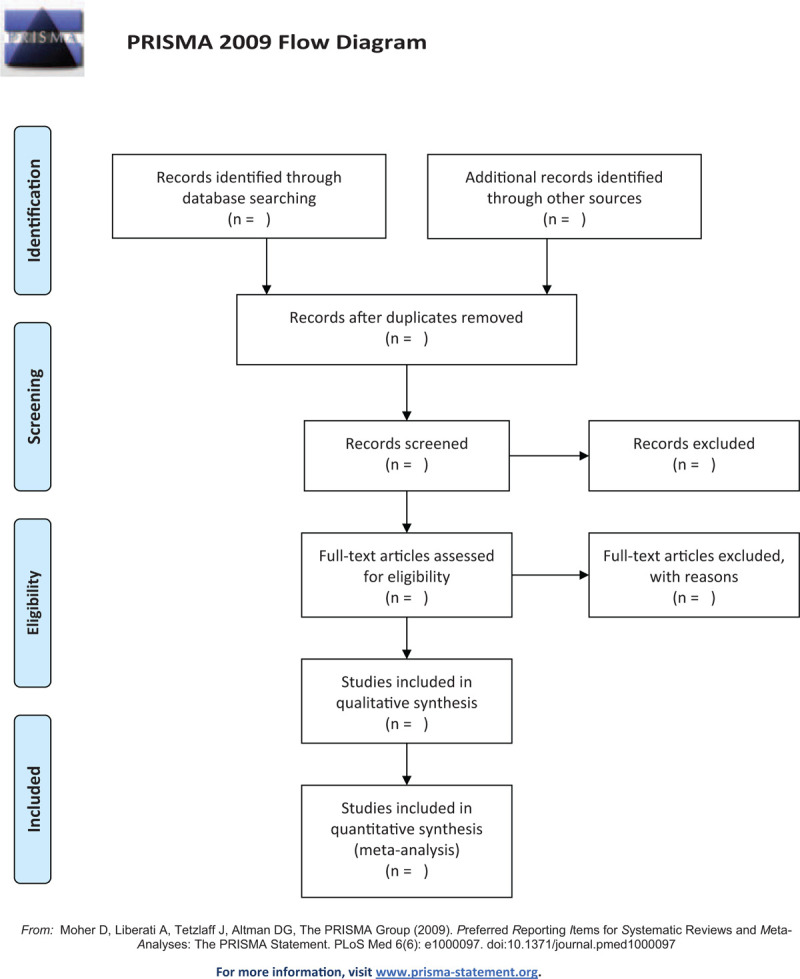
Flow diagram of studies search and selection.

Two medical workers (SQS and YGC) engaged in the clinical treatment of AF will conduct a literature search respectively. We will strictly follow the pre-established inclusion and exclusion criteria for the preliminary screening and secondary screening of the literature. At the same time, the 2 researchers will independently extract the data, and finally submit it to the third researcher (YHH) conducts the comparison. If there is any fallacy or disagreement, the third researcher will make a judgment. If there are missing data or information in the literature, we will try to contact the author by email or phone to complete it. The literature retrieval process of our study will use the PRISMA flowchart, as shown in Figure 1. For each excluded study, reasons for exclusion will be given.

The main data or materials extracted from the literature data of this study include: the title of the paper, the first author, the year of publication, the sample size of each arm, the precautionary measures, the age, the course of treatment, the follow-up time, the related items of the literature quality evaluation and the related outcome indicators.

### 2.5 Assessment of study quality

This study will use Cochrane Handbook 5.1.0 risk bias tool for the quality assessment.^[14]^ The tool includes evaluation in 7 areas: random sequence generation, allocation hiding, blinding of participants and personnel, blinding of outcome measurers, incomplete result data, selective reports, and other biases. The risk of bias in each area will be judged as low, high, or unclear. In a study, if the risk in all areas is low, then the overall risk of bias will be classified as low risk. If the risk in one or more areas is high, then the overall risk of bias will be classified as high risk. Otherwise, it will be classified as an unclear risk.

### 2.6 Data synthesis and analysis

In the statistical analysis, for continuous variables, the weighted mean difference after treatment will be used as the effect size, and the 95% confidence interval (credibility integral) of weighted mean difference will be calculated.^[15]^ As corresponding, count data will use odds ratio and the 95% confidence interval of odds ratio as the effect size of statistical data. In this study, the *χ*^2^ test will be used to test the heterogeneity of the included studies. *P* > .05 and *I*^*2*^ < 50% will be used as the criteria for heterogeneity test. When the above 2 statistical conditions are met, it indicates that the combined effect size performed has good homogeneity, and the fixed effects model is used. If after the heterogeneity test, as long as one of them does not meet, it indicates that the homogeneity of the combined effect size is not ideal, and the random effects model needs to be used for statistics.^[16]^

Stata 14.0 software (STATA Corporation, Lakeway, Texas, USA) will be used to draw the network evidence relationship diagram, forest diagram, rank probability diagram, funnel diagram, and corresponding statistics.^[17]^ The node splitting method (node-splitting measurement) will be used for consistency test.^[18]^ If the difference is not statistically significant (*P* > .05), it indicates that the results of direct comparison and indirect comparison are consistent, then Stata14.0 will be used to test each closed loop for consistency. In this study, the cumulative ranking probability of each treatment scheme will be calculated by using surface under the Cumulative ranking (SUCRA). The larger the value of SUCRA, the better the effect of this intervention.^[19]^

### 2.7 Assessment of publication biases and sensitivity analysis

The Begg test will be used to analyze the potential publication bias. If *P* > .05, it means that there is no publication bias. If *P* < .05, it means that there is publication bias.^[20]^ When conducting sensitivity analysis, after excluding the research with low quality, high weight, and different results from other studies, we will calculate the combined statistics and compare with the combined statistics before the exclusion. If the 2 results are the same, then result of meta-analysis is stable, conversely, the results would be unstable.

### 2.8 Ethics and dissemination

It is not necessary for ethical approval because it is based on published studies. The protocol will be disseminated in a peer-reviewed journal or presented at a topic-related conference.

## 3 Discussion

As a common clinical arrhythmia disease, AF is accompanied by high prevalence, high disability, and high mortality. In recent years, studies have suggested that WXG can selectively affect atrial muscle action potentials,^[21]^ which have a significant inhibitory effect on AF induced by acetylcholine, through inhibiting matrix remodeling after atrial ablation. At the same time, clinical studies confirmed that WXG combined with low-dose amiodarone to maintain sinus rhythm therapy after radiofrequency in patients with AF can improve heart function, reduce drug side effects, and obtain more clinical benefits. Our network meta-analysis will conduct a detailed summary and analysis of WXG combined with AADs in the treatment of AF to find the best treatment plan. A particular advantage of our study will be to increase the safety analysis of various antiarrhythmic treatments, and we will collect data on drug-related side effects in our study to assess the tolerance of different treatments. The results of this meta-analysis may help physicians determine the best treatment options for AF patients.

## Author contributions

**Conceptualization:** Shuqing Shi, Qiulei Jia.

**Data curation:** Shuqing Shi, Yuguang Chu.

**Methodology:** Yuguang Chu.

**Project administration:** Yuanhui Hu.

**Writing – original draft:** Shuqing Shi, Yuguang Chu.

**Writing – review & editing:** Shuqing Shi, Qiulei Jia.
